# Rehabilitation for people with dementia: a multi-method study examining knowledge and attitudes

**DOI:** 10.1186/s12877-020-01940-x

**Published:** 2020-12-09

**Authors:** Kate E. Laver, Maria Crotty, Lee-Fay Low, Lindy Clemson, Craig Whitehead, James McLoughlin, Kate Swaffer, Monica Cations

**Affiliations:** 1 Flinders Health and Medical Research Institute, Flinders University, GPO Box 2100, Adelaide, SA 5001 Australia; 2 Faculty of Medicine & Health, The University of Sydney, Sydney, New South Wales Australia; 3 College of Nursing and Health Sciences, Flinders University, Adelaide, South Australia Australia; 4 Dementia Alliance International, Adelaide, Australia; 5 College of Education, Psychology and Social Work, Flinders University, Adelaide, South Australia Australia

**Keywords:** Rehabilitation, Dementia, Health services, Attitudes, Ageing

## Abstract

**Background:**

People with dementia are not routinely offered rehabilitation services despite experiencing disability associated with the condition and accumulating evidence for therapies such as exercise, occupational therapy, and cognitive or physical rehabilitation. It is important to understand the needs and preferences of people with dementia regarding rehabilitation services. The aim of this study was to explore thoughts and beliefs about rehabilitation amongst people with dementia and their families.

**Methods:**

Interviews with people with dementia and family members regarding their experience of care following diagnosis and their attitudes and beliefs about rehabilitation for dementia. Surveys with older people with cognitive impairment and/or a diagnosis of dementia to determine preferences for services and understanding of rehabilitation programs.

**Results:**

Interviews with 13 participants (*n* = 6 people living with dementia with mean age 60 and *n* = 7 care partners) revealed gaps in care post diagnosis. People reported having to seek out services and frequently sought out services which were rehabilitative in nature. Survey data (*n* = 91 participants, average age 82) showed that most people had heard of rehabilitation (92%) or had experience of rehabilitation (49%) at some point. There was a wide range of services identified as being beneficial. Rehabilitative interventions including case management, exercise and memory strategies were considered desirable.

**Conclusions:**

People with dementia report having a wide variety of needs. There are gaps following diagnosis where people with dementia report having to seek out their own services. Some interview participants (who tended to be younger) clearly articulated the need for tailored interventions which maximised independence and quality of life. Survey participants, who were on average older, reported that they would participate in individually applicable rehabilitative therapies if they were available.

## Background

The global prevalence of dementia is rising sharply and is expected to exceed 131 million by 2050 [1]. Improvements in awareness and monitoring can mean that people with dementia are diagnosed earlier in their disease course with relatively fewer cognitive and functional impairments [1]. Such advancements present opportunities for early intervention and the delivery of services to maximise independence and community participation throughout the disease course, as recommended in Clinical Practice Guidelines [2, 3]. These recommendations are based on evidence that non-pharmacological interventions including exercise, cognitive rehabilitation, and occupational therapy can slow the progression of cognitive and functional decline [4, 5], facilitate goal achievement [6], and delay institutionalisation [7].

Despite these recommendations, multidisciplinary rehabilitation programs are not a part of the usual treatment pathway for dementia. This is unlike other chronic neurological conditions (like multiple sclerosis) and acquired brain injuries, for which rehabilitation is more accepted in the clinical pathway [8]. People with dementia are also less likely to be included in rehabilitation programs for comorbid acute conditions [9, 10]. Academics [11–13] and consumer groups [14] have argued that the impairments caused by dementia and their secondary effects (e.g. stigma, reduced community participation) can be conceptualised as social disability and should therefore trigger access to rehabilitation supports. This is consistent with the World Health Organization definition of rehabilitation as a holistic approach to chronic disease management, rather than simply relevant to recovery after physical injury [15].

Health professionals have questioned the potential goals and benefits of rehabilitation in dementia care, citing concerns about the potential for meaningful outcomes for this population and the ability for people with dementia to master new learning [16]. However, several studies have demonstrated that people with dementia can benefit from rehabilitation similarly to people without dementia [17–20], especially with the assistance of technology [21, 22]. Achieving these similar outcomes does necessitate greater resource use, and health professionals cite this as one reason for their reluctance to refer or accept people with dementia for rehabilitation [16, 23]. In addition, whether rehabilitative and palliative approaches to care can coexist remains an active matter of debate [16, 24–26]. As a result alternative terms are commonly used to refer to arguably rehabilitative services, including ‘reablement’ or ‘restorative’ care [27].

While there are increasing calls for rehabilitation to be available for people with dementia, there has been less exploration of how these services might be delivered or which elements are of highest priority. Dementia Alliance International, an advocacy body run by people living with dementia, have argued that access to rehabilitation is a human right under the Convention on the Rights of Persons with Disabilities [28]. Research has not been conducted to examine how people with dementia and their care partners conceptualise delivery of rehabilitation services, how this would be different to current care delivery, or which elements are of highest priority. Understanding the end user’s understanding of and preferences for rehabilitative services will be important guiding clinical and policy decision making.

The aim of this study was to examine understanding of rehabilitation among people with dementia and their families, assess whether this approach is desirable and/or consistent with their beliefs about best-practice care provision, and examine their perspectives about how, when, and why rehabilitation should be delivered.

## Method

### Design

A interpretive descriptive approach [29] was applied to this work, with a multi-methods design including qualitative interviews and quantitative surveys. We had originally planned to conduct qualitative interviews only, but difficulty recruiting older people with dementia to be interviewed meant that the sample included predominantly those with young onset dementia (YOD) and care partners. Given that people with YOD are known to have different experiences and priorities than both older people with dementia and care partners [30, 31], a quantitative survey of older people with dementia was subsequently conducted to examine the external validity of the themes identified from interviews. Both parts of this research were conducted during the rollout of a national disability insurance scheme for which people with YOD are eligible but older people with dementia are not. We report our qualitative results according to the COnsolidated criteria for REporting Qualitative research (COREQ) [32].

### Participants and data collection

#### Qualitative interviews

A convenience sample of 13 interview participants were recruited through social media, dementia advocacy organisation newsletters, email invitations through existing networks of the research team, and snowball sampling. Eligible participants had a diagnosis of dementia or provided informal (i.e. unpaid) care to a person with dementia, spoke fluent English, were living in the community (i.e not in residential aged care) and were able to provide informed consent. Dementia diagnoses were self-reported and were not verified by the research team. One-off semi-structured qualitative interviews were conducted by MCa, who has many years of experience working with people with dementia in both clinical and research settings. She had only worked directly with one participant, who had contributed expertise to a previous research project. Interviews were private and confidential, conducted either in the participant’s home or over the phone. The interview asked the person with dementia to describe their experiences of care following diagnosis, attitudes towards rehabilitation in general, and beliefs about rehabilitation for people with dementia. Where participants had favourable attitudes toward rehabilitation for dementia, they were asked to consider how they would prefer these services be delivered. Questions were open to reduce the risk of imposing the interviewer’s assumptions and beliefs (which have been published [12]). Interviews were conducted from June 2017 to September 2018 and lasted between 60 and 90 min. Participants were provided with a small honorarium in recognition of their time contributing to the research. All interviews were audio recorded and transcribed verbatim. Recruitment and interviews continued until the authors conducting analysis recognised that few new ideas were being raised by participants, suggesting that saturation had been reached [33]. However, there are likely undiscovered themes because older people with dementia are underrepresented in our sample.

#### Quantitative surveys

Quantitative survey participants were recruited via an aged care assessment service and from a Geriatric Evaluation and Management (GEM) ward in a large hospital in metropolitan Adelaide. Patients admitted to the GEM ward were eligible to complete the survey if they had a score of 19–24 (indicating dementia or mild cognitive impairment) on the Mini Mental State Examination [34], usually resided in the community, and were fluent in English. Patients recruited via the aged care assessment service were eligible if cognitive impairment or dementia was recorded in their assessment, they spoke fluent English, and they consented to their contact details being passed to the research team. Dementia diagnoses in these assessments required written verification from a medical professional. Participants recruited from the GEM wards completed the survey verbally in person from their room, while participants recruited from the aged care assessment service completed the survey over the phone. AS or SB administered all surveys and did not deviate from the written questions.

The survey, which was specifically designed for this research project, (Supplementary Table 1) examined the external validity of the themes identified in qualitative interviews. The research team, who include specialist rehabilitation physicians, allied health professionals, and consumer experts, designed the survey to capture participants’ understanding of and preferences for rehabilitation services based on what was learned during interviews. The survey was piloted with five people with dementia and refined to improve the clarity of the question wording. It first gathered demographic details from the person, sought to understand their main symptoms, and asked whether they had heard of or received rehabilitation before. Participants were asked to provide a brief definition of rehabilitation which was recorded verbatim. Participants were then asked to rate the extent to which they agreed with a series of questions about their satisfaction with post-diagnosis services (for example ‘My doctor has put me in contact with other services to help me’) on a five-point scale from ‘Strongly Disagree’ to ‘Strongly Agree’. Finally, the person was asked to identify how likely they would be to use a range of potential rehabilitation services right now (if it were free) on a five point scale from ‘Definitely Would Not Use’ to ‘Definitely Would Use’. As much of Australia’s health and aged care system is publicly funded, participants were informed that the services would come at no direct cost to them. All participants received the same list and items included on this list were deliberately varied to include services delivered by a range of professionals.

### Data analysis

Qualitative interview data analysis was conducted using an inductive approach, consistent with guidance by Ezzy [35]. Analysis began with two authors (KEL and MCa) individually reading each transcript to develop a thematic analysis plan. KEL then conducted an iterative process of reading and rereading each transcript and conducted line-by-line coding to develop a coding system. For example, the statement *“Because there was no one who really pointed us in the direction of, look – you can go to this service or that service or do this or that, you know. And I had no idea what was out there for dementia other than some people are in nursing homes”* reported by ‘Carer 6’ was coded as “gaps after diagnosis”. The coding system was validated by MCa by applying it to two transcripts. Categories that were generated using this coding system were linked where appropriate. For example, the code “inequities with other conditions” was linked with “human rights”. The final categories and links were discussed and refined by KEL and MCa until final agreement was reached [36]. There were no major differences in the categories identified in phone or in-person interviews. Written reflections created by the interviewer were included in analysis to consider the ways in which her personal experiences and biases may have influenced the results. We include quotations here as representative examples of the themes identified in the data.

Quantitative survey data were analysed using SPSS software version 26 [37]. Results are presented descriptively using percentages and averages. Definitions of rehabilitation were grouped into categories using a content analysis approach whereby MCa and KEL individually read each response and grouped them according to common phrases and latent meanings. These groupings were then refined and agreed during discussion. Qualitative and quantitative data was triangulated following the procedure described by Foster [38], whereby pertinent results within each method were compared and their conceptual similarities and differences noted.

### Ethics

This study was approved by the Southern Adelaide Clinical Human Research Ethics Committee (HREC/16/SAC/454).

## Results

Six people living with dementia and seven care partners of people living with dementia participated in a qualitative interview. Interview participants with dementia included four women and were aged 60 years on average (range 50 to 67 years). All the care partner participants were women. Six provided care to their male spouse with dementia and one provided care to her mother. The people with dementia they cared for were aged 65 years on average (range 60 to 79 years). Dementia diagnoses included Alzheimer’s disease (*n =* 6), frontotemporal dementia (*n =* 6) and Lewy Body disease (*n =* 1), diagnosed on average 6 years prior to the interview (range 1–9 years). Nine of the 13 people with dementia (interview participants or care recipients of interview participants) received a disability or aged care pension while the remaining four relied on private income. Eleven received funding for services under the aged care (*n* = 7) or disability systems (*n* = 4).

Demographic details of the 91 people with cognitive impairment or dementia who completed surveys appear in Table 1. They were aged 82 years on average (range 65 to 97 years) and were mostly male (68.1%). The qualitative and quantitative participant groups did not overlap; that is, none of the interview participants also completed a survey.

**Table 1:** Quantitative survey sample characteristics

Item (n^a^)	ẋ (SD) or *n* (%)
Age (90)	82.4 (9.4)
Female (91)	29 (31.9)
Born outside Australia (91)	28 (30.8)
Language other than English (91)	8 (8.8)
Aboriginal and/or Torres Strait Islander (91)	2 (2.2)
Income source (86)	
Full pension	65 (75.6)
Part pension	15 (16.5)
Paid employment	2 (2.3)
Self-funded retiree	65 (75.6)

^a^*n* < 91 indicates missing data

Four main themes could be identified in the interview data, that were then further explored in the quantitative surveys: (a) experiences with post-diagnostic care, referring to the existing context that shapes their views toward rehabilitation; (b) highly conceptual understanding of rehabilitation; (b) mixed views about rehabilitation for people with dementia in a general or theoretical sense, and; (c) engagement in rehabilitative services.

### Experiences with post-diagnostic care

Most interview participants reported that they had been diagnosed with dementia by a specialist medical practitioner (neurologist, psychiatrist or geriatrician), but few participants were linked with dementia specific supports beyond the appointment.

*“It was medical, very medical …. … I wasn’t referred to anybody … .apart from see you in six months …. There wasn’t a brochure from Alzheimer’s Australia in his office, nothing” (PWD1)*

*“They gave me a piece of paper … it was basically the dementia nurses within the hospital system, as it were, and suggested that I ring one of those locally with no real indication of why I’d want to ring them” (PWD4).*

Some had been linked with services that, at the time, were available only for people with YOD to help identify suitable community supports. These services were highly valued by those who could access them and missed by those who could not. However, there were some limitations to the service even when it was available as some participants felt that more continuity of care was required. Other issues related to difficulty communicating needs with the key worker and turnover of staff.

*“The key worker actually rang me once or twice to see if there was anything. But it’s one of these catches of is there anything we can do, but you don’t know what they can do so there’s no real way of answering that, or asking those questions without that sort of knowledge” (PWD4).*

Participants in the study were receiving a variety of services including community-based groups run through councils or carer organisations. Several people had utilised social and day respite programs. In most cases participants had initiated these services themselves through their own research.

*“I found my own merry way to the Alzheimer’s Association” (PWD2).*

*“I’m pretty resourceful myself, so I started hunting down things on Google and started out hunting down various peak bodies and all of that sort of thing” (Carer8).*

This process of discovery was described as a “battle’ for an outsider to the health and aged care system. One participant reported that they turned to people they knew in health and aged care through their social networks to help understand the system.

*“It was only because I went through someone I knew. Otherwise I think I would still be struggling” (PWD1).*

*“You are literally pushing at the system every step of the way. Why on earth does the government make it this difficult?” Carer8.*

People with dementia who completed the quantitative survey reported similarly inconsistent experiences with care (Table 2), with 36.9% of participants reporting that they would not know who to call if they wanted to access a service. More than 45% reported that they did not know how the aged care system works.

**Table 2:** Experiences with services (included *n* = 84, missing *n* = 7)

	Strongly disagree	Disagree	Undecided	Agree	Strongly Agree	I don’t know
I have access to the help and support I need to do the things that are important to me	1 (1.2)	11 (13.1)	4 (4.8)	51 (60.7)	12 (14.3)	5 (6.0)
My doctor has put me in contact with other services to help me	13 (15.5)	19 (22.6)	6 (7.1)	37 (44.0)	4 (4.8)	5 (6.0)
I know who to call if I want to access a service	10 (11.9)	21 (25.0)	3 (3.6)	33 (39.3)	11 (13.1)	6 (7.1)
I understand how the aged / community care system works	20 (23.8)	19 (22.6)	9 (10.7)	32 (38.1)	2 (2.4)	2 (2.4)
I need more help to keep doing the things I like to do	7 (8.3)	31 (36.9)	2 (2.4)	26 (31.0)	14 (16.7)	4 (4.8)

### Understanding of rehabilitation

Interview participants overall had a comprehensive understanding of rehabilitation. They understood it to be focused on enhancing independence and quality of life, and that it was individualised and tailored to the needs and goals of the individual.

*“Rehabilitation is, my understanding, is getting back to some sort of semblance of what might have been a normal lifestyle” Carer10.*

However, a coherent understanding of rehabilitation was less common among the people with dementia who completed the quantitative survey as presented in Table 3. While most (92.3%) had heard of rehabilitation, more than 30% could not provide a definition. Only 26.4% of all definitions demonstrated an strong understanding of the enablement aim of rehabilitation (for example, “*A support team to assess difficulties and help you achieve goals*”, “*Helping people get back onto their feet, back into the world*”), while others were more generic (e.g. “*To help you get better*”) or specifically referenced illness or injury (e.g. “*Time to recover after being ill or injured*”).

**Table 3:** Understanding of rehabilitation

Item (n^a^)	*n* (%)
Heard of rehabilitation (91)	84 (92.3)
Experience with rehabilitation (90)	44 (48.9)
Definition of rehabilitation (91)	
Enablement-focussed definition	24 (26.4)
Generic recovery	19 (20.9)
Recovery from illness/injury	12 (13.2)
Care when ill/dependent	6 (6.6)
Specific therapies and providers	2 (2.2)
I don’t know / missing / unclear	28 (30.8)

^a^*n* < 91 indicates missing data

### Views about rehabilitation for people with dementia

Most interview participants agreed with taking a rehabilitative approach to care for people with dementia. Indeed all agreed that it was important for dementia care to be individualised, to focus on the person’s strengths and what the person can do and maintaining that focus where possible and promoting quality of life from early dementia symptoms until the end.

*“Doesn’t mean that I’ll get completely better. It means I can maintain independence in as many areas as possible for as long as possible” (PWD1).*

*“I see it as providing people with coping mechanisms to keep their lives as positive and fulfilling for as long as they can” (PWD2).*

*“Of course with dementia they can’t ever be the person that they were before, but, you know, I can see this even as rehabilitation because it’s getting him to be – keeping him the best that he can be” (Carer9).*

While some participants could relate this approach of maximising independence and quality of life with the term rehabilitation, others struggled with the terminology. Often, participants had perceptions of rehabilitation which involved recovery to pre-morbid function. People used examples such as recovering after a broken leg or recovering from substance addiction.

*“I’m never going to be rehabilitated to back where I was prior to my diagnosis. However, there are certain skills and certain abilities that I have now post-diagnosis that I want to maintain, to keep intact. And though I’m not going to call it rehabilitation I like the word reablement.” (PWD3).*

*“The term sounds a bit strange partly because it’s something that, I think maybe it’s one of those terms that needs a new word because realistically rehabilitation to regain skills that you’re losing is not going to happen” (PWD4).*

One participant was cautious that offering rehabilitation services may offer false hope:

*“It gives a false hope … And that’s very dangerous. It can be very dangerous. Moreso, for the family …. Probably just calling it ‘therapy’ or just something – keeping it deliberately vague could probably be the most helpful (PWD5).*

Whereas others reported that a major problem with current services is that they didn’t offer any hope:

*“A major issue is that there isn’t any hope given. That is a major thing. If the people who diagnose can’t actually give particularly younger people who are usually in the mild stages still some hope that they can perhaps continue to work with support, they can continue to do their hobbies and still continue with their interests and whatever but they’re not given any …” (Carer7).*

There was also discussion about the timing of rehabilitation and when it might be most appropriate. One participant suggested that there was a role for rehabilitation for people in residential care.

*“Everyone gives up on them when they go into residential care, and particularly when their mobility becomes impaired, they’re just left and they just go downhill like a rocket” (Carer6).*

Whereas another participant felt that rehabilitation would not be beneficial for people with more severe symptoms but was appropriate in the early stages.

*“I feel like if someone was early stage dementia, that it’s probably a lot more applicable because it may be in helping them pre-plan and adjust” (Carer8).*

Participants described access to rehabilitation as being a human rights issue and felt that there were inequities between the care offered for people with dementia and the care offered for people with other conditions.

*“We just want to be supported to live our lives, whatever our lives are and I think that’s a fairly basic human right to do that” (PWD1).*

*“I was absolutely gobsmacked that there were no designated rehab type services for people with dementia, which is basically a neurological disability, that what it is, we know. And why it was viewed and treated differently to every other neurological disability I could not understand.” (Carer6).*

Inequities in accessing services were also present for people living in regional areas. Travelling to access services was considered a great challenge.

*“All she could suggest that was available would be a monthly morning coffee meeting which was only 80 kilometres away” (PWD4).*

Two participants described how telehealth options could overcome this challenge.

*“When therapies start coming online of any kind, it’ll be so welcome” (PWD5).*

Participants who were care partners spoke of the need for rehabilitation programs to incorporate carers. This could either be through participating in activities together or using the time and opportunity to connect carers and provide each other with peer support.

*“I think what you have to remember is the carer. So, you could have the best rehabilitative, you know, things and programs, but without looking after the carer it’s not going to work, you know” (Carer9).*

### Seeking out services which are rehabilitative in nature

Most qualitative interview participants described how they had sought out activities and services which they felt would enhance their independence and quality of life or that of the person with dementia they provided care for. Their choice of activities and services suggest that rehabilitative approaches focussing maintaining independence are sought after following a diagnosis of dementia.

*“I’ve made up my own little – I guess you might call it program” (PWD5).*

*“I’ve just recently signed up for (local council run) drawing classes … And I cannot tell you how much I love this group …. I could just feel my brain like really working hard to do that. And I’ve really loved it” (PWD2).*

However, quantitative survey participants provided mixed responses when asked to indicate how likely they would be to use a range of rehabilitative services (Table 4). While system navigation and transport services were highly sought-after, support to engage in employment and volunteering and help to find attend social groups were viewed less favourably.

**Table 4:** Rehabilitation services (*n* = 91)

	Definitely would not use	Probably would not use	Undecided	Probably would use	Definitely would use	I don’t know	I already use this
Case manager	6 (6.6)	18 (19.8)	4 ((4.4)	19 (20.9)	22 (24.2)	4 (4.4)	18 (19.8)
Group exercise classes	41 (45.1)	11 (12.1)	0 (0)	18 (19.8)	14 (15.4)	1 (1.1)	6 (6.6)
Counselling	27 (29.7)	2 (24.2)	1 (1.1)	22 (24.2)	12 (13.3)	0 (0)	7 (7.7)
Mobility aids	4 (4.4)	2 (2.2)	1 (1.1)	3 (3.3)	7 (7.7)	0 (0.0)	74 (81.3)
Home exercise programs	12 (13.2)	15 (16.5)		26 (28.6)	19 (20.9)	4 (4.4)	15 (16.5)
Memory strategies	29 (31.9)	18 (19.8)	5 (5.5)	18 (19.8)	15 (16.5)	4 (4.4)	2 (2.2)
*Those with memory loss (n = 50)*^*a*^	*8 (16.0)*	*9 (18.0)*	*5 (10.0)*	*13 (26.0)*	*11 (22.0)*	*2 (4.0)*	*2 (4.0)*
Help to find and attend social groups	34 (37.4)	18 (19.8)	1 (1.1)	16 (17.6)	11 (12.1)	1 (1.1)	10 (11.0)
Meal delivery service (*n* = 43)	14 (32.6)	5 (11.6)	5 (11.6)	6 (11.6)	4 (9.3)	0 (0)	43 (20.9)
Speech pathology	50 (54.9)	14 (15.4)	3 (3.3)	12 (13.2)	8 (8.8)	4 (4.4)	0 (0.0)
*Those with word finding difficulties (n = 30)*	*9 (30.0)*	*5 (16.7)*	*1 (3.3)*	*6 (20.0)*	*6 (20.0)*	*3 (10.0)*	*0 (0.0)*
One-on-one exercise class	25 (27.5)	17 (18.7)	1 (1.1)	27 (29.7)	10 (11.0)	1 (1.1)	10 (11.0)
Help at home (*n =* 43)	2 (4.7)	0 (0.0)	0 (0.0)	5 (11.6)	3 (7.0)	1 (2.3)	32 (74.4)
Support groups	46 (50.5)	19 (20.9)	2 (2.2)	15 (16.5)	3 (3.3)	3 (3.3)	3 (3.3)
*Those with memory loss (n = 50)* ^*a*^	*17 (34.0)*	*13 (26.0)*	*2 (4.0)*	*10 (20.0)*	*3 (6.0)*	*2 (4.0)*	*3 (6.0)*
Employment or volunteering	59 (64.8)	12 (13.2)	2 (2.2)	9 (9.9)	3 (3.3)	2 (2.2)	4 (4.4)
*Those wanting to work or volunteer but not able to (n = 9)*	*3 (33.3)*	*2 (22.2)*	*0 (0.0)*	*3 (33.3)*	*1 (11.1)*	*0 (0.0)*	*0 (0.0)*
Transport	14 (15.4)	10 (11.0)	1 (1.1)	16 (17.6)	27 (29.7)	2 (2.2)	21 (23.1)
*Those with driving difficulties (n = 19)*	*0 (0.0)*	*0 (0.0)*	*0 (0.0)*	*3 (16.7)*	*11 (57.9)*	*1 (5.6)*	*4 (22.2)*
System navigator	16 (17.6)	12 (13.2)	2 (2.2)	9 (9.9)	36 (39.6)	3 (3.3)	6 (6.6)

^a^Includes those who indicated that they forget things, lose things, and have difficulty orienting to time

## Discussion

This multi-methods study draws together findings from interviews with people with dementia and family members of people with dementia and surveys with older people with cognitive impairment or dementia. Our aim was to understand the attitudes and beliefs of these cohorts towards rehabilitation and to explore preferences for different types of services. People who responded to our advertisements inviting people with dementia to talk about rehabilitation tended to be younger (with average age 60 years) and due to our method of recruitment were likely more familiar with dementia services and progressive in their views about dementia care. Most participants had a good understanding of rehabilitation as maximising independence and quality of life and felt that this was an important goal of care for people with dementia. However, not all participants agreed that rehabilitation was the most appropriate term given that it was often associated with recovery-oriented programs. In contrast, participants who completed the survey were approached via health services and offered a broader range of views on rehabilitation. Survey respondents were also considerably older (average age 82 years). Although this cohort were familiar with the concept of rehabilitation, they tended to consider rehabilitation programs as focussed on regaining lost function (such as after injuring oneself). Family members of people with dementia were also supportive of therapies to enhance quality of life and maintain function. Survey respondents identified a wide range of services that they may use, and these depended on their needs and goals. Despite the differences in sample selection, age, and beliefs about rehabilitation we found that people from both cohorts felt that gaps in care exist and there is a need for individualised services which promote independence and quality of life.

The World Health Organisation describes rehabilitation as a holistic approach to optimise function and reduce the experience of disability. Furthermore, the Organisation describes rehabilitation as being necessary not just for those recovering from injury or illness but for those with detriments in function linked to ageing. This more contemporary description of rehabilitation would be unfamiliar to the general public and it would not have previously occurred to many of our survey participants that rehabilitation for disability associated with ageing or dementia could be beneficial. Providing survey participants with education around what can be offered and the associated benefits and then asking them about their needs may have resulted in different answers. Furthermore, without direct experience of a therapy or intervention it can be difficult to make a judgement about whether that therapy or intervention may be beneficial. Several of our interview participants described seeking out rehabilitative services or therapies and experiencing benefits.

The findings of this study have some similarities to our earlier work in which we interviewed health professionals to understand their attitudes and beliefs about rehabilitation for people with dementia [16]. Health professionals similarly felt that the term ‘rehabilitation’ implied that the person participating was recovering from an event, illness, or injury (such as a stroke). Hence, health professionals considered programs which were described as “rehabilitation” to be less appropriate for people with dementia than programs which were described as being “reablement” even though the content of the program may be the same. Health professionals and people with dementia both raised concerns about hope and how to delicately balance offering hope while not giving false hope. Working with hope presents challenges for health professionals working in a wide range of clinical areas and is not exclusive to the field of dementia care [39].

There is increasing evidence that rehabilitative interventions are of benefit for people with dementia. Rehabilitation has been shown to help people with dementia achieve their nominated goals [6], can delay functional decline [4] and may delay admission to residential care [7]. However, the diversity of needs and preferences for rehabilitation amongst people with dementia poses challenges for health service planners. How can rehabilitative services be made available to the people who need them within an environment of scarce resources and a large (and increasing) population of people with dementia? Restricting services based on age, symptoms, or severity of impairment creates inequities and creates ethical challenges [14]. Yet in many countries the availability of services does depend on age and severity of dementia symptoms. It is also important to note that rehabilitation interventions for other conditions, like stroke, are cost-effective as they lead to reduced admissions to hospital and delayed admission to residential care [40]. Unfortunately, there is an absence of research to date examining longer term consequences of rehabilitation interventions for people with dementia (and/or their families).

### Strengths and limitations

Our use of multi methods with different sampling strategies and populations provided us with the opportunity to compare and contrast. However, use of social media to recruit interviewees introduce a sampling bias in favour of younger people with dementia, with lower levels of cognitive impairment, and higher digital literacy. People were included in the interviews if they had a self-reported diagnosis of dementia and we did not verify the diagnosis. These participants gave rich and detailed descriptions of their process of being diagnosed with dementia and we have no reason to doubt the accuracy of their diagnosis. These participants also opted into an interview about rehabilitation for people with dementia, and so were found to have a personal interest in the topic. It is also possible that the interviewer’s views about the potential value of rehabilitation for people with dementia [12] influenced the interview outcomes, despite efforts to reduce this bias. Most interviews were conducted over the phone by necessity due to participants living in other states of Australia. It is possible that phone interviews may result in some loss to the richness of information.

Survey participants were approached via a health service and therefore offer a more representative sample of older people with cognitive impairment or dementia. However, a larger population including those not accessing health services and those with more severe cognitive impairments would have increased generalisability. Recruiting from one single health service also limits generalisability. Another limitation is that changes in the aged care environment have been made since we completed data collection, including the introduction of new quality standards and an upgraded platform for accessing services. We are unable to comment on whether these changes resulted in improved services for people with dementia and easier navigation of services.

## Conclusion

Our research shows that rehabilitative interventions and therapies are valued by a wide range of people with dementia and their families although the types and level of services desired varies. Research is now required to determine how rehabilitation can be offered in a way that reaches those in need, that leads to beneficial outcomes for the person and that is cost effective.

## Supplementary Information

**Additional file 1.**

## Data Availability

The datasets used and analysed during the current study are available from the corresponding author on reasonable request.

## Abbreviations

PWD: Person with Dementia
SD: Standard deviation
YOD: Young onset dementia
