# Supplementary material for: Rehabilitation for people with dementia: a multi-method study examining knowledge and attitudes
Source: BMC Geriatr. 2020 Dec 9;20:531. doi: 10.1186/s12877-020-01940-x (PMC7727118; doi:10.1186/s12877-020-01940-x)
Supplement: Supplementary file 1 — Additional file 1. [file 12877_2020_1940_MOESM1_ESM.pdf]

The Rehab-D Study

HREC Approval Number: HREC/16/SAC/434

Rehab-D

Thank you for deciding to participate!

All you need to do is:

1. Fill in the questionnaire on the next pages

2. Put the questionnaire into the enclosed envelope and put it into a post box. You do not need to put a stamp on it.

You can ask a family member or friend to help you fill out this questionnaire. Please make sure the responses reflect **your thoughts**.

Start the questionnaire on the next page.

Your day-to-day difficulties

First, what are the main things that have caused you difficulty lately? Your family member or friend might be able to help you with this.

Please choose all that apply by placing a tick in the box ☑:.

- ☐ 1. Forgetting things, like people's names and appointment times
- ☐ 2. Having trouble finding the right words
- ☐ 3. Feeling down or blue
- ☐ 4. Confusion about what day or time it is
- ☐ 5. Finding it difficult to read
- ☐ 6. Having trouble handling cash (e.g. figuring out how many coins to give)
- ☐ 7. Falling over or worrying that you might fall over
- ☐ 8. Having trouble driving or not being able to drive anymore
- ☐ 9. Eating too much or not eating enough
- ☐ 10. Getting angry or upset more easily
- ☐ 11. Aches and pains
- ☐ 12. Putting something down and then forgetting where you've put it
- ☐ 13. Wanting to work or volunteer but not being able to
- ☐ 14. Feeling lonely
- ☐ 15. Something else (please write below):

____

____

____

____

____

Rehabilitation

Next, we want to learn about your understanding of the word 'rehabilitation'.

Have you ever heard of 'rehabilitation' before?

☐ No
☐ Yes
☐ I'm not sure

Have you ever received rehabilitation care before?

☐ No
☐ Yes
☐ I'm not sure

If yes, when and for what condition?

____

____

____

Please give us a brief explanation about what you think rehabilitation care is. If you're not sure, just write "I don't know".

____

____

____

____

____

Your experiences

Now we would like to ask a few questions about your experiences with community / aged care services. Circle the number that describes how strongly you agree or disagree with the following statements.

1. I have access to the help and support I need to do the things that are important to me					
Strongly disagree	Disagree	Undecided	Agree	Strongly agree	I don't know
2. My doctor has put me in contact with other services to help me					
Strongly disagree	Disagree	Undecided	Agree	Strongly agree	I don't know
3. I know who to call if I want to access a service					
Strongly disagree	Disagree	Undecided	Agree	Strongly agree	I don't know
4. I understand how the aged / community care system works					
Strongly disagree	Disagree	Undecided	Agree	Strongly agree	I don't know
5. I need more help to keep doing the things I like to do					
Strongly disagree	Disagree	Undecided	Agree	Strongly agree	I don't know

Your opinion of service ideas

Next, we are going to list some different services that you we think might be useful. We want to know your opinion of these ideas.

Please tell us how likely you would be to use each service AT THE MOMENT if it was free and available in your area by circling your response. If you're not sure, circle 'I don't know'.

1. A case manager or care coordinator to help you choose and access services						
Definitely would not use	Probably wouldn't use	Undecided	Probably would use	Definitely would use	I don't know	I already use this
2. Group exercise classes at a gym, park, dance studio or clinic						
Definitely would not use	Probably wouldn't use	Undecided	Probably would use	Definitely would use	I don't know	I already use this
3. Counselling or therapy at a local clinic						
Definitely would not use	Probably wouldn't use	Undecided	Probably would use	Definitely would use	I don't know	I already use this

Please tell us how likely you would be to use each service AT THE MOMENT if it was free and available in your area by circling your response. If you're not sure, circle 'I don't know'.

4. In-home mobility aids like rails, walking frames or chairs						
Definitely would not use	Probably wouldn't use	Undecided	Probably would use	Definitely would use	I don't know	I already use this
5. At-home exercise program written by a physiotherapist						
Definitely would not use	Probably wouldn't use	Undecided	Probably would use	Definitely would use	I don't know	I already use this
6. Strategies to managing memory loss or other thinking difficulties						
Definitely would not use	Probably wouldn't use	Undecided	Probably would use	Definitely would use	I don't know	I already use this
7. Help to find and attend social groups or activities that you enjoy						
Definitely would not use	Probably wouldn't use	Undecided	Probably would use	Definitely would use	I don't know	I already use this
8. A meal delivery service						
Definitely would not use	Probably wouldn't use	Undecided	Probably would use	Definitely would use	I don't know	I already use this

Please tell us how likely you would be to use each service AT THE MOMENT if it was free and available in your area, by circling your response. If you're not sure, circle 'I don't know'.

9. Assessment and advice to help with word finding or other language changes						
Definitely would not use	Probably wouldn't use	Undecided	Probably would use	Definitely would use	I don't know	I already use this
10. One-on-one exercise classes with a trainer, at a gym, park or clinic						
Definitely would not use	Probably wouldn't use	Undecided	Probably would use	Definitely would use	I don't know	I already use this
11. Help at home (e.g. with cleaning, home maintenance)						
Definitely would not use	Probably wouldn't use	Undecided	Probably would use	Definitely would use	I don't know	I already use this
12. Support groups with other people with memory loss and other thinking changes						
Definitely would not use	Probably wouldn't use	Undecided	Probably would use	Definitely would use	I don't know	I already use this

Please tell us how likely you would be to use each service AT THE MOMENT if it was free and available in your area, by circling your response. If you're not sure, circle 'I don't know'.

13. Support to access work or volunteering opportunities						
Definitely would not use	Probably wouldn't use	Undecided	Probably would use	Definitely would use	I don't know	I already use this
**14. Assistance with transport**						
Definitely would not use	Probably wouldn't use	Undecided	Probably would use	Definitely would use	I don't know	I already use this
**15. Someone to contact you regularly and help you access more support if you need it**						
Definitely would not use	Probably wouldn't use	Undecided	Probably would use	Definitely would use	I don't know	I already use this

Rehab-D

About you

Lastly, we want to know a bit about you.

1. How old are you?

   _______ years

2. What is your gender?

   ☐ Male
   ☐ Female
   ☐ Other

3. Were you born in Australia?

   ☐ Yes
   ☐ No, I was born in ______________________

4. Is English your first language?

   ☐ Yes
   ☐ No, my first language is ______________________

5. Do you identify as Aboriginal or Torres Strait Islander?

   ☐ No
   ☐ Yes, Aboriginal
   ☐ Yes, Torres Strait Islander
   ☐ Yes, both Aboriginal and Torres Strait Islander

6. What income do you receive?

   ☐ Full pension
   ☐ Part pension
   ☐ Paid employment
   ☐ Self-funded retiree

Thank you!

That is the end of the questionnaire. Thank you very much for your answers. Please place the questionnaire in the enclosed envelope and put it into a postbox.

What will happen to information about me?

The information you provide to us in the survey is anonymous. There is no information that could identify you. Your information will only be used for the purpose of this research project, and you have the right to request access to your survey responses after you send them back.

The data from this study will be owned by Flinders University. We will keep your anonymous survey for a period of at least 7 years, after which it will be destroyed. If you return your questionnaire and later decide that you don't want us to use it, you can contact us on the details below.

This research project has been approved by the Human Research Ethic Committee (HREC) of the SA Department for Health and Ageing. If you have any concerns or complaints, you can contact them on (08) 8226 7235 or email HealthHumanResearchEthicsCommittee@sa.gov.au.
